# Effects of Temperatures and Heatwaves on Occupational Injuries in the Agricultural Sector in Italy

**DOI:** 10.3390/ijerph20042781

**Published:** 2023-02-04

**Authors:** Chiara Di Blasi, Alessandro Marinaccio, Claudio Gariazzo, Luca Taiano, Michela Bonafede, Antonio Leva, Marco Morabito, Paola Michelozzi, Francesca K. de’ Donato, on behalf of the Worklimate Collaborative Group

**Affiliations:** 1 Department of Epidemiology Lazio Regional Health Service, ASL ROMA 1, 00147 Rome, Italy; 2 Occupational and Environmental Medicine, Epidemiology and Hygiene Department, Italian Workers’ Compensation Authority (INAIL), 00143 Rome, Italy; 3 Institute of Bioeconomy, National Research Council (IBE-CNR), 50019 Florence, Italy

**Keywords:** work-related injuries, occupational injuries, agricultural sector, temperatures, heat waves, timeseries studies

## Abstract

The effects of heat on health have been well documented, while less is known about the effects among agricultural workers. Our aim is to estimate the effects and impacts of heat on occupational injuries in the agricultural sector in Italy. Occupational injuries in the agricultural sector from the Italian national workers’ compensation authority (INAIL) and daily mean air temperatures from Copernicus ERA5-land for a five-year period (2014–2018) were considered. Distributed lag non-linear models (DLNM) were used to estimate the relative risk and attributable injuries for increases in daily mean air temperatures between the 75th and 99th percentile and during heatwaves. Analyses were stratified by age, professional qualification, and severity of injury. A total of 150,422 agricultural injuries were considered and the overall relative risk of injury for exposure to high temperatures was 1.13 (95% CI: 1.08; 1.18). A higher risk was observed among younger workers (15–34 years) (1.23 95% CI: 1.14; 1.34) and occasional workers (1.25 95% CI: 1.03; 1.52). A total of 2050 heat-attributable injuries were estimated in the study period. Workers engaged in outdoor and labour-intensive activities in the agricultural sector are at greater risk of injury and these results can help target prevention actions for climate change adaptation.

## 1. Introduction

Temperatures across Europe and the Mediterranean basin are constantly rising, with the last ten summers registering above-average values, as reported by Copernicus Climate Services [1]. Summer 2022 registered a record +2.8 °C above the climatological average (1991–2020) and +0.4 °C higher than the previous year on record. As reported in the latest IPCC report, climate change is a matter of fact, and extreme climatic events, and increasing temperatures have been shown to have adverse impacts on human health in terms of increased mortality and morbidity with different impacts depending on age, gender, and socioeconomic characteristics, and will continue in the future with more frequent occurrences [2]. There is a growing body of emerging studies on the impact of climate change on the occupational sector, and the negative consequences concern capacity and costs in the production process, health injuries, and workers’ health [3,4].

Adverse effects of heat and climate change on human health have been documented in numerous epidemiological studies all over the world [5,6] and some of them posed the question of the impact of extreme heat on workers’ health [7,8,9]. In fact, workers employed in specific occupational sectors working outdoors can be particularly exposed to extreme events and physical fatigue for prolonged periods of time, which can lead to heat stress [10,11,12,13], with consequences not only on productivity and occupational costs [14,15,16], but also on work capacity [17], with possible consequences on occupational injuries [18]. Moreover, a series of surveys were conducted [19,20,21] and found that the perception of heat-related risk in workplaces is underestimated by workers, so it is crucial to strengthen their awareness of the risks and define adequate prevention strategies.

Evidence of the increasing risk of occupational injuries associated with high temperatures has been found in different geographical settings [22,23,24,25,26,27,28,29,30]. More recently, reviews have not only confirmed the association between occupational injuries and heath exposure but also summarized the evidence on vulnerability factors and sectors most at risk [31,32,33,34,35,36,37,38].

In Italy, several studies have been conducted on heat-related occupational injuries. A study conducted in Tuscany evaluated the association between heat and hospital admissions due to work-related accidents and found an increase in admissions on days with high apparent temperature [39]. A study conducted in three Italian cities (Rome, Milan, and Turin) showed an association between high temperatures and occupational injuries among workers employed in the construction, transportation, and energy sectors [40]. Most recently, Marinaccio et al. conducted a national study on temperature-related occupational injuries and found a significant relative risk of 1.17 (95% CI: 1.14–1.21) for increases in mean temperature above the 75th percentile and highlighted differences in risk estimates among economic sectors [22]. Moreover, Gariazzo et al. focused on occupational injuries related to heat waves and high temperatures in the construction sector and found significant relative risks for the particular type of workers, production processes, and specific activities performed before the accident [41]. Nevertheless, an Italian study on the evaluation of both occupational risks and impacts in the agricultural sector has not been carried out.

The aim of this study is to estimate the association between daily air temperatures and occupational injuries in the agricultural sector at the municipal level in Italy using national compensation claims. Furthermore, the study estimates the relative risk and attributable injuries for heat and heatwave exposures identifying individual vulnerability factors among agricultural workers.

## 2. Materials and Methods

### 2.1. Workers’ Compensation Data

Data on 150,422 work-related injuries occurring in Italy between 2014–2018 were extracted from the Italian workers’ compensation authority (INAIL) archives. Occupational injury claims related to the agricultural sector were selected and daily counts of events were calculated for each of the 8068 municipalities of Italy. Anonymization procedures were applied in order to ensure privacy.

Data includes information on gender, age at injury, professional qualification (labourer, self-employed, occasional), and duration of leave, considered as a proxy of severity of the injury.

Occupational injuries occurring while travelling (road accidents) and injuries occurring among individuals aged less than 15 years and over 85 were excluded. Data were also stratified by different variables (gender; age group: 15–34, 35–60, 61+; professional qualification: labourer, self-employed, occasional; duration of leave: 0–14, 15–29, 30–60, 61+ days); working process: crop production and harvesting, plant breeding, livestock farming and breeding, land preparation, auxiliary preparation, forestry, other).

### 2.2. Meteorological Data

Daily mean air temperature data for the study period were retrieved from ERA-5 Land climate reanalysis data [42] available from the Copernicus Climate data Store (CDS) and were considered as exposure variable.

For each of the 8068 Italian municipalities, the daily mean air temperature was calculated as the average mean temperature of all the grid cells included in the spatial domain of the municipality weighted by the area of inclusion.

A time series dataset of daily injuries and daily mean temperatures for each municipality for the entire 5-year study period (2014–2018) was constructed.

### 2.3. Statistical Analysis

Analyses of this work were produced with three different methodologies but with the common background of Distributed Lag Non-linear Model (DLNM) approach to take into account both the potential non-linear shape of the dose-response curve and the delayed effect of the exposure on the outcome [43,44].

The relationship between mean air temperature and injuries was modelled with a B-spline with one internal knot at the 50th percentile of region-specific temperature distributions, and the lag response with a categorical variable (lag window 0–2). An over-dispersed Poisson generalized regression model was used for the analyses, and time-varying covariates were fitted:

- summer population decrease (a 3-levels variable with value “2” for the 2-week period around 15 August; “1” from 16 July to 31 August with the exception of the aforementioned 2-week period; “0” elsewhere);
- public holidays (a 4-levels variable with value “1” on isolated days; “2” on Christmas, Easter and New Year’s Day; “3” on the days surrounding Christmas, Easter, and New Year’s Day; “0” elsewhere);
- a four-way interaction by municipality, year, month, and day of the week to control for long-term time trends and seasonality.

### 2.4. Effect Estimates

To estimate the exposure-response curve and the relative risks, a two-stage approach was considered. Firstly, for each of the 19 Italian regions (Valle d’Aosta region was excluded due to limited observations), specific over-dispersed Poisson generalized linear regression models were applied, while, in the second stage, the regional estimates were combined to obtain an overall dose–response curve, and effect-estimates by applying a multivariate meta-analytical regression [45].

Results for high temperatures are reported as the Relative Risk (RR) and 95% Confidence Intervals (95% CI) of work-related injuries in the agricultural sector for increases in mean temperature between the 75th and 99th percentile.

Effect modification was evaluated by stratifying the analysis by age group (15–34, 35–60, and 61+ years), injury severity (defined as the duration of leave in days and categorized as 0–14, 15–29, 30–60, and 61+ days), professional qualification (labourer, self-employed, occasional) and working process (crop production and harvesting, plant breeding, livestock farming and breeding, land preparation, auxiliary preparation, forestry, other).

### 2.5. Impact Estimates (Attributable Injuries)

In order to account for the impact of heat on occupational injuries in the agricultural sector, the number of attributable injury cases associated with the same temperature interval and relative 95% empirical Confidence Interval (95% eCI) were estimated, according to the methodology described in Gasparrini and Leone [44]. Moreover, the number of attributable cases by age, injury severity, and professional qualification variables were also estimated.

### 2.6. Heatwaves

To evaluate the effect of extreme events in summer, the analysis was restricted to the warm months (May to September), and the risk of occupational injury for heatwave days was estimated.

Firstly, heatwaves (HWs) were defined as three or more consecutive days of mean air temperature above the municipality-specific 90th percentile in the warm months. Secondly, the regional risk of injury on heatwave days, compared to non-heatwave days was estimated. Similarly to the previous analysis, the model was adjusted for day of the week, a two-way interaction term between municipality and year, and controlled for seasonal time trends with a spline modelled on the days of the warm period. Thirdly, regional estimates were meta-analysed to obtain an overall RR and relative 95% CI, and the attributable number of injuries occurring during HWs was calculated.

All analyses were performed using the R statistical software version 4.1.3 (http://R-project.org, accessed on 16 September 2022).

## 3. Results

**Figure 1:**
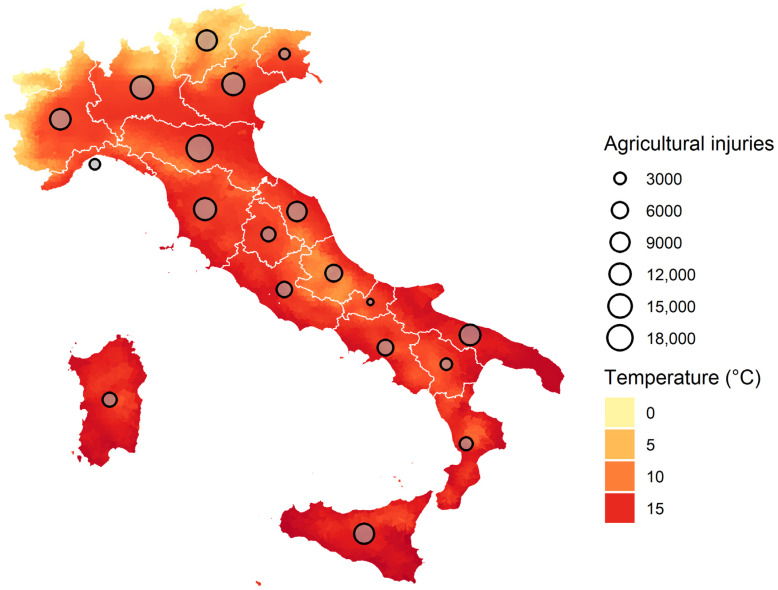
Daily mean air temperature and occupational injuries in the agricultural sector in Italy in the period 2014–2018. Air temperature is expressed at municipal resolution, while injuries are at the regional level.

During the study period (2014–2018) a total of 150,422 occupational injuries in the agricultural sector were reported in the 19 Italian regions (Table 1), with a decreasing trend over time both for annual and summer counts. The same trend was observed in each region (Table A1). Figure 1 shows the total number of occupational injuries for each region during the study period with the highest percentage of injuries in the Northern regions of Emilia-Romagna, Lombardia, Veneto, Toscana in the Centre and Puglia in the South (regional values are reported in Appendix A Table A1). The gender distribution of injuries is predominantly male (78%) reflecting the higher proportion of males employed in the agricultural sector in Italy. The majority (over 50%) of injuries occurred in the 35–60 years old age group in all the regions, while in a few of them (Friuli-Venezia Giulia, Lombardia, Puglia, and Sicilia) a higher number of injuries was observed among the youngest age group (15–34 years). As for the duration of leave, considered as a proxy of injury severity, 30% of the agricultural injuries were non-severe (<14 days leave) with a declining trend by increasing severity. Injury claims by professional qualification were heterogeneous among regions, with more than 50% of total injuries occurring among self-employed workers, with the highest proportion in Abruzzo (80%) and Molise (84%), and lowest in Calabria (16%), where the occasional workers had the highest proportion of injury claims (around 46% compared to a national average of 14%). Labourer injury claims were around 27% nationally, ranging from 12% in Abruzzo and Molise to 42% in Lombardia.

Figure 1 illustrates the mean air temperature in the study period at the municipal level showing a North–South gradient with higher temperatures in the Southern regions. The mean air temperature in the five-year period was of 12.9 °C, with the highest value in 2018 and the lowest in 2016 (Table 1 and Appendix A Table A2). The complex orography and its geographical location in the Mediterranean influence the climate of Italy and its regions. Mean temperatures in the Northern regions vary from 6.4 °C in Trentino-Alto Adige, 13.5 °C in Central regions, and 15.6 °C in the South, with the maximum value in Puglia (16.8 °C). Similarly, the percentiles considered in the analysis range from 12.6 °C to 22.4 °C for the 75th percentile and from 21.5 °C to 29.8 °C for the 99th, respectively in the coldest (Trentino-Alto Adige) and in the warmest (Puglia) region (Table 1).

Considering heatwaves during the warm season (May to September), around 15% of the days were identified as heatwaves, with an annual average of 24 HWs per year ranging between 5 in 2014 and 38 in the summer of 2015. The average temperature during a heatwave was of 24.9 °C.

**Figure 2:**
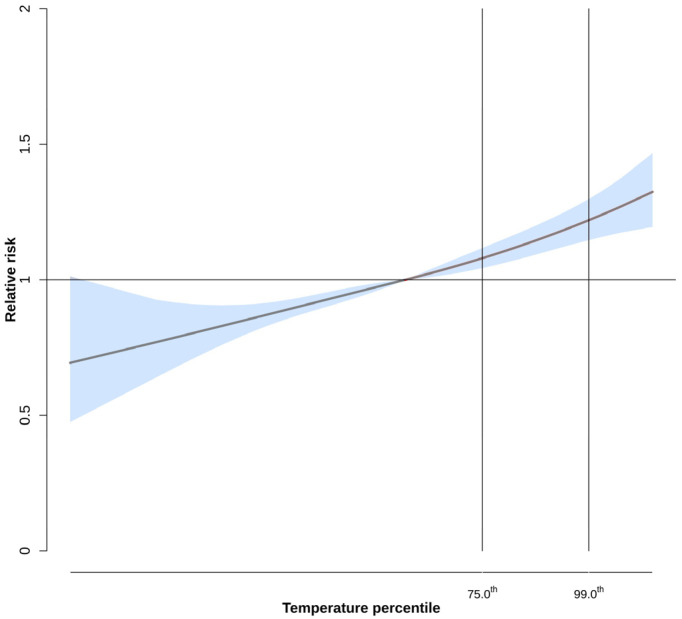
Meta-analytical exposure-response curve between daily mean air temperature and occupational injuries in the agricultural sector in Italy in the period 2014–2018. Estimates are expressed as Relative Risks (thick lines) and 95% confidence bands.

Figure 2 shows the exposure-response curve of the association between daily mean air temperature and the risk of agriculture-related injuries.. The vertical lines represent the mean temperature percentile interval (75th and 95th) between which the risk of heat-related occupational injuries has been estimated. The figure shows a linear association between temperature and agricultural injuries with increasing risks as temperatures rise.

**Figure 3:**
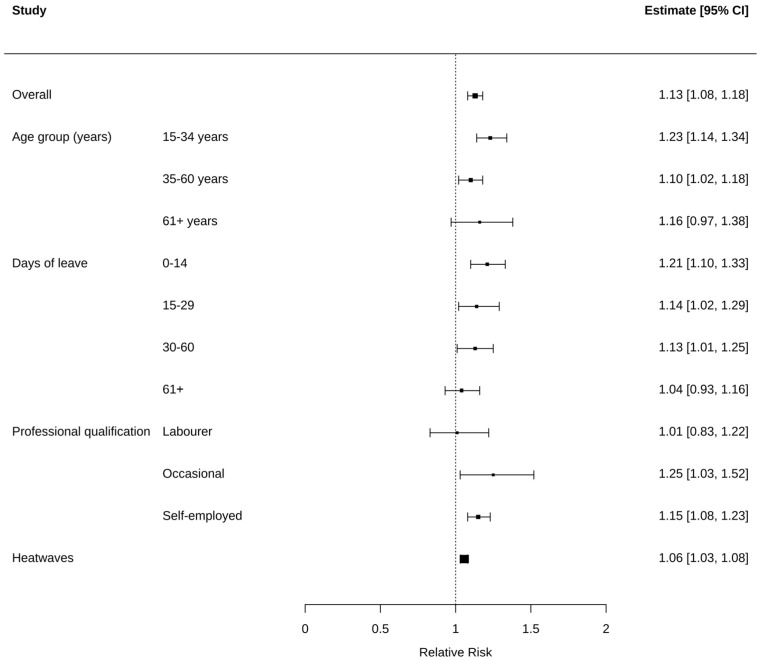
Relative Risks (and 95% confidence intervals) of work-related injuries in the agricultural sector for increases in daily mean temperature between 75th to 99th percentile (period 2014–2018). Square size represents the robustness of the estimates.

The cumulative relative risks (RR) of work-related injuries in the agricultural sector, associated with an increase in temperature between the 75th to 99th percentile, are reported in Figure 3. The overall RR was 1.13 (95% CI 1.08–1.18) and a greater risk of injury was observed among young workers aged from 15 to 34 years (RR 1.23, 95% CI: 1.14–1.34), occasional and self-employed workers (RR 1.25, 95% CI: 1.03–1.52 and RR 1.15, 95% CI: 1.08–1.23, respectively). Furthermore, agricultural workers have a greater risk of experiencing a non-severe (RR 1.21, 95% CI: 1.10, 1.33) or a mild injury (RR 1.14, 95% CI: 1.02, 1.29) than severe ones (RR 1.13, 95% CI: 1.01, 1.25 for 30–60 days of leave and RR 1.04, 95% CI: 0.93, 1.16 for more than 60 days). Considering working processes, a significant risk was found for workers carrying out land preparation (RR 1.18, 95% CI: 1.08, 1.30) and other agricultural processes (RR 1.16, 95% CI: 1.05, 1.27) (Table A4).

The risk of work-related injuries in the agricultural sector during HWs (3 or more consecutive days above the warm season 90th percentile) was 6% higher than on non-HW days (Figure 3).

Table 2 shows the number of injuries attributable to increases in daily mean air temperature between the 75th to 99th percentile. Over the entire 5-year study period, a total of 2050 heat-attributable injuries were estimated with an average of 410 per year. Considering worker subgroups, the greatest impact was observed among those aged 35–60 years and considering employment type, as expected, the self-employed category had the greatest number of heat-related injuries.

## 4. Discussion

This study explored the relationship between daily mean air temperature and the risk of occupational injuries among agricultural workers in Italy from 2014 to 2018. A relative risk of 1.13 (95% CI 1.08–1.18) for exposures between the 75th and 99th percentile of air temperature in the whole study period was found.

Several studies have evaluated the association between air temperature and occupational injuries, with the majority of these considering heat stress and HWs, but few of them focused on the agricultural sector [31,32,33,34,35,36,37,38]. Although all studies found a positive association between high temperatures and work-related injuries, comparisons are difficult because of differences in study design, statistical techniques, HW definitions, geographical or climatological settings, and sectors/activities included.

The physiological link between heat exposure and workers concerns both health and productivity [11] and depends on individual characteristics [10] as well as outdoor working conditions [46], that can be, however, mitigated by practices like hydration, work-time shifting, work-rest cycles and ventilated clothing [15,47,48,49]. In this context, the recent Italian Worklimate project has developed a heat stress forecasting system for different outdoor working scenarios [50], developing informative and training material for employers and workers to help raise awareness and prevent heat stress and injuries among workers (https://www.worklimate.it/en/, accessed on 17 November 2022).

An increasing risk of injuries for agricultural workers has been previously shown in Italy, especially in the North, both in the autonomous province of Trento in the first decade of 2000s [26], and in the Po River Valley in the second one [51]. Similarly, a study conducted in Spain, which has both similiar climatic conditions and agricultural activities to Italy, showed the highest percent risk difference (almost 30%) of injury associated with extreme temperatures in the 99th percentile versus the minimum occupational injury percentile among agricultural workers [23]. In Australia, studies conducted in different cities and regions confirm a significant risk of heat-related injuries among agricultural workers [29,52]. A study conducted in Brisbane reported a RR of 1.91 (95% CI: 0.72–5.03) for “agriculture, forestry and fishing” for exposures to high temperatures (99th percentile) while in Adelaide, the RR for “agriculture, forestry, fishing and hunting” category was even higher (4.01 (95% CI: 1.24–12.9) [29]. A study conducted in Washington State, USA [27], found an odds ratio of 1.10 (95% CI 1.01, 1.20) for outdoor traumatic injuries among agricultural workers due to apparent temperature values above 33 °C compared to lower ones (<25 °C). Findings from our study, in terms of risk estimates and the positive association between heat and occupational injuries in the agricultural sector are consistent with the evidence in the literature and meta-analytical results [31].

Although several studies on occupational injuries investigated the effect modification of the association with high temperatures, few of them focused on risk factors for agricultural workers. Riccò et al. reported the highest odds ratio in very young workers (<20 years old) related to >95th percentile of mean air temperature with a fluctuating trend among other age groups [51]. The estimates of this work report higher risks in the 15–34 and 61+ years age groups, respectively of 1.23 and 1.16, statistically significant only in the first case and consistently with the variability of Riccò’s trend. A meta-analysis reported a higher risk (RR: 1.009, *p*-value: < 0.001) for young workers (age <35 years), possibly attributable to inexperience [31] but, on the other hand, there is evidence of higher risks among elderly workers, due to physiological mechanisms [11,53] and comorbidities [54]. In Italy, a greater risk for the under 35s is reported by both Marinaccio et al. [22] and Gariazzo et al. [41], probably due to an underestimation of the risk or a lack of training on specific risks [21]. In 35–60 year old workers, although a lower risk was found, the highest impact in terms of the number of attributable injuries was estimated, as the greatest proportion of workers are in this age group, suggesting the need to enhance prevention measures and awareness campaigns for both workers and employees. When considering the severity of injuries, only one case-crossover study, previously mentioned, on agricultural workers in Washington State [27], found a greater risk in mild-severe and severe injuries (25–29, 30–33, 34 or more days of leave) which is in contrast to findings from our study, in which a decreasing risk at increasing severity of injury was observed. In the context of professional qualification of agricultural workers, a higher risk was estimated for occasional and self-employed workers, and self-employers also showed the highest impact (attributable injury cases). It is plausible that both these categories could be the less trained and experienced, in the first one because of the temporary nature of work, in the second one due to the absence of colleagues with more experience to learn from.

The definition of HWs varies among studies and sensitivity analyses suggested to not directly compare studies that use different definitions [6]. However, two studies investigated the effect of HWs on occupational injuries in Australia, both defining HWs as three consecutive days with maximum temperature over 35 °C, and obtained contrasting results. In fact, the first one, conducted in Adelaide [55], found a positive incidence rate ratio of 1.45 (95% CI 1.13–1.86) for “agriculture, forestry and fishing” while a second study [56] found a non-significant relative risk of 0.98 (95% CI 0.62–1.54) for “agriculture, forestry, fishing and hunting” workers. Contrasting results came out also when considering the severity of HWs defined by a newly proposed metric of heatwave severity, the Excess Heat Factor (EHF) index [57], with negative risks for low and high-severity HW days and positive for moderate ones. The definition chosen for HWs considered in this study is consistent with previous studies conducted in Italy and with the definition used in the Italian Heat Health Watch Warning System (HHWWS) [58,59].

The strengths of this work lie in the coverage of the outcome, which includes injury claims at the national level in the agricultural sector and on the high spatial resolution of the exposure. Moreover, both injuries and temperature data are detailed at the municipal level. For the first time, this study provides estimates of attributable injuries in the agricultural sector by age, days of leave, professional qualification and HWs. However, it is also worth mentioning the limitations of the impossibility of including the irregular workers not registered in the INAIL database, underestimating the number of injuries, and a great heterogeneity in agricultural activities and processes carried out between regions.

In summary, the study shows that high temperatures are a significant risk factor for occupational injuries, with stringer effects among the young, occasional, or self-employed workers.

In coming years we can expect that climate change and a warming climate will enhance the adverse impacts on occupational health and work productivity around the world [2,12]. A recent study estimated that Under RCP8.5 by 2100, global GDP declines by 1.4% due to heat stress [4]. It was estimated that in Italy, the labour productivity loss will more than double in 20 years from 300 million dollars in 2010 to 650 in 2030 [59]. Furthermore, it has been estimated that in Southern Europe in 2030 the total hours of work lost due to heat stress will double with respect to 1995 and for Italy, the same result is expected in the agricultural sector [3]. Specific adaptation and protective strategies to protect workers in the context of climate change need to be promoted. Warning systems for specific occupational settings, improving thermal characteristics of working environments, reducing physical activity in work settings, use of protective clothing, hydration, and cooling spaces need to be implemented and provided as well as research on monitoring heat exposure and physiological heat stress and evaluating preventive actions need to be enhanced. Future studies in the occupational sector should address region-specific area and individual worker risk factors and develop sector-specific response measures, in order to define more effective prevention strategies.

## 5. Conclusions

Heat has a significant impact on occupational injuries in the agricultural sector and adequate prevention measures need to be introduced to reduce risks and respond to future climate change. The results of this study could be useful in the awareness of such problems and fruitful in implementing prevention actions and working conditions in the agricultural sector, which is one of the sectors at highest risk due to climate change.

## Figures and Tables

**Table 1:** Descriptive statistics of occupational injuries in the agricultural sector, mean temperature and heatwaves in Italy in the study period (2014–2018).

		Full Period		Summer (May–September)	
		Frequency	Percentage	Frequency	Percentage
Overall		150,422	100	66,025	100
Year	2014	33,362	22.2	14,555	22.0
	2015	31,846	21.2	14,002	21.2
	2016	30,033	20.0	13,126	19.9
	2017	28,453	18.9	12,342	18.7
	2018	26,728	17.8	12,000	18.2
Sex	Male	117,874	78.4	51,339	77.8
	Female	32,548	21.6	14,686	22.2
Age group (years)	15–34	27,085	18.0	12,103	18.3
	35–60	94,122	62.6	41,243	62.5
	61+	29,215	19.4	12,679	19.2
Days of leave	0–14	45,421	30.2	20,636	31.3
	15–29	36,413	24.2	16,001	24.2
	30–60	36,054	24.0	15,507	23.5
	61+	32,534	21.6	13,881	21.0
Professional qualification	Labourer	41,377	27.5	18,896	28.6
	Occasional	21,687	14.4	9690	14.7
	Self-employed	87,345	58.1	37,434	56.7
		**Annual average**		**Summer (May-September)** **Average**	
Termperature °C	Mean	13.0		19.7	
	Min	−24.7		-5.8	
	1°	−4.6		7.0	
	25°	7.2		16.6	
	50°	12.9		19.7	
	75°	19.0		23.2	
	99°	28.0		29.1	
	Max	35.0		35.0	
				**N (%)**	**Average Temperature °C**
Heatwaves *	Yes	-	-	118 (15.4)	24.9°C
	No	-	-	647 (84.6)	18.7°C

* Heatwaves are defined as three or more consecutive days of mean temperature above the 90th percentile in summer months (May–September).

**Table 2:** Relative Risks (and 95% confidence intervals) and number of heat attributable injuries (and 95% empirical confidence intervals) in Italy for increases in mean temperature between the 75th to 99th percentile in the full period 2014–2018.

		RR (95% CI)	Attributable Injuries	95% eIC	
Overall		1.13 (1.08–1.18)	2050	1632	2455
Age group (years)	15–34	1.23 (1.14–1.34)	396	346	446
	35–60	1.10 (1.02–1.18)	1258	1024	1487
	61+	1.16 (0.97–1.38)	464	405	521
Days of leave	0–14	1.21 (1.10–1.33)	739	618	852
	15–29	1.14 (1.02–1.29)	578	492	660
	30–60	1.13 (1.01–1.25)	485	404	565
	61+	1.04 (0.93–1.16)	337	260	409
Professional qualification	Labourer	1.01 (0.83–1.22)	748	664	831
	Occasional	1.25 (1.03–1.52)	405	352	460
	Self-employed	1.15 (1.08–1.23)	1051	801	1300
Heatwaves *		1.06 (1.03–1.08)	608	−72	1237

* Heatwaves are defined as 3 or more consecutive days of mean temperature above the 90th percentile in summer months (May–September).

## Data Availability

Data cannot be shared due to privacy restrictions.

## Appendix A

**Table A1:** Descriptive statistics of occupational injuries in the agricultural sector by region, in Italy in the study period (2014–2018).

		Year											
		2014		2015			2016		2017			2018	
Regions	Total	Freq	%	Freq		%	Freq	%	Freq		%	Freq	%
Piemonte	10,710	2414	22.5	1040		22.0	968	20.2	882		18.6	821	16.7
Lombardia	13,771	1414	23.1	1281		20.9	1271	20.0	1132		18.8	1079	17.2
Trentino-Alto Adige	10,533	1089	22.8	984		20.5	916	18.9	893		18.6	958	19.3
Veneto	13,114	1298	22.0	1262		21.5	1182	19.9	1103		18.7	1084	17.9
Friuli-Venezia Giulia	2444	243	20.6	247		22.4	221	20.4	216		18.9	205	17.8
Liguria	2466	247	22.4	233		21.9	204	19.9	197		18.9	169	16.9
Emilia-Romagna	19,299	1989	22.2	1872		21.6	1760	19.7	1603		18.4	1668	18.1
Toscana	12,794	1215	22.3	1174		21.8	1045	19.7	996		18.5	1006	17.6
Umbria	4301	418	22.4	399		21.9	360	19.2	295		18.6	317	18.0
Marche	9574	886	22.7	922		21.2	816	20.1	723		18.9	699	17.0
Lazio	5367	511	23.3	513		22.4	383	18.5	434		18.9	406	16.9
Abruzzo	6590	612	22.4	611		22.0	532	19.8	517		19.3	455	16.4
Molise	1619	174	24.1	149		20.0	136	20.5	118		17.5	136	17.9
Campania	5631	513	22.2	510		19.7	517	20.6	505		19.0	488	18.5
Puglia	11,136	899	20.4	984		20.5	968	20.9	850		18.9	905	19.2
Basilicata	2909	308	22.4	290		21.2	258	19.6	251		20.9	201	15.8
Calabria	3726	345	20.4	319		20.1	368	22.1	375		20.8	279	16.6
Sicilia	10,043	817	20.2	836		19.7	856	20.9	856		20.1	817	19.2
Sardegna	4395	500	24.5	376		20.5	365	19.3	396		19.4	307	16.3
		**Age Group (Years)**						**Professional Qualification**					
		**15–34**		**35–60**		**31+**		**Labourer**		**Occasional**		**Self-Employed**	
**Regions**	**Total**	**Freq**	**%**	**Freq**	**%**	**Freq**	**%**	**Freq**	**%**	**Freq**	**%**	**Freq**	**%**
Piemonte	10,710	2043	19.1	6023	56.2	2644	24.7	1880	17.6	548	5.1	8281	77.3
Lombardia	13,771	3111	22.6	8523	61.9	2137	15.5	5783	42.0	696	5.1	7292	53.0
Trentino-Alto Adige	10,533	1917	18.2	6115	58.1	2501	23.7	1867	17.7	593	5.6	8073	76.6
Veneto	13,114	2455	18.7	7825	59.7	2834	21.6	4186	31.9	566	4.3	8361	63.8
Friuli-Venezia Giulia	2444	555	22.7	1458	59.7	431	17.6	844	34.5	152	6.2	1447	59.2
Liguria	2466	451	18.3	1668	67.6	347	14.1	633	25.7	169	6.9	1664	67.5
Emilia-Romagna	19,299	3302	17.1	11,361	58.9	4636	24.0	5022	26.0	2872	14.9	11,404	59.1
Toscana	12,794	2319	18.1	7759	60.6	2716	21.2	4885	38.2	1068	8.3	6839	53.5
Umbria	4301	676	15.7	2638	61.3	987	22.9	1295	30.1	446	10.4	2558	59.5
Marche	9574	932	9.7	5486	57.3	3156	33.0	2025	21.2	537	5.6	7012	73.2
Lazio	5367	1072	20.0	3399	63.3	896	16.7	1477	27.5	696	13.0	3191	59.5
Abruzzo	6590	651	9.9	4264	64.7	1675	25.4	799	12.1	542	8.2	5249	79.7
Molise	1619	170	10.5	1157	71.5	292	18.0	188	11.6	74	4.6	1357	83.8
Campania	5631	808	14.3	4154	73.8	669	11.9	1413	25.1	850	15.1	3368	59.8
Puglia	11,136	2435	21.9	7675	68.9	1026	9.2	2358	21.2	4782	42.9	3996	35.9
Basilicata	2909	440	15.1	2061	70.8	408	14.0	748	25.7	752	25.9	1409	48.4
Calabria	3726	785	21.1	2663	71.5	278	7.5	1398	37.5	1718	46.1	610	16.4
Sicilia	10,043	2291	22.8	6757	67.3	995	9.9	2913	29.0	4451	44.3	2677	26.7
Sardegna	4395	672	15.3	3136	71.4	587	13.4	1663	37.8	175	4.0	2557	58.2

**Table A2:** Descriptive statistics of air temperature and heatwaves by region, in Italy in the study period (2014–2018).

	Temperature °C				Heatwaves *	
Regions	Mean	SD	Percentiles		N	Mean Temperature °C
			75th	99th		
Piemonte	11.1	8.0	17.4	26.6	116	23.4
Lombardia	12.1	8.0	18.5	28.0	117	24.8
Trentino-Alto Adige	6.4	7.9	12.6	21.5	109	18.5
Veneto	12.7	8.0	19.1	28.6	116	25.4
Friuli-Venezia Giulia	11.8	7.7	18.0	26.9	116	24.0
Liguria	13.2	6.4	18.6	25.4	114	23.7
Emilia-Romagna	13.7	7.7	19.8	29.0	118	26.5
Toscana	13.9	6.9	19.5	27.5	122	25.5
Umbria	13.4	7.2	19.1	27.9	123	25.9
Marche	13.9	7.1	19.6	28.1	119	26.0
Lazio	13.8	7.0	19.5	27.7	128	25.6
Abruzzo	12.0	7.3	17.7	26.9	117	24.0
Molise	12.9	7.1	18.6	27.2	117	24.9
Campania	14.7	6.7	20.2	27.9	125	25.9
Puglia	16.8	6.7	22.4	29.8	123	28.2
Basilicata	13.7	7.2	19.5	28.6	118	26.0
Calabria	15.3	6.3	20.5	27.9	117	25.8
Sicilia	16.2	6.4	21.6	28.9	112	26.9
Sardegna	15.9	6.4	21.4	28.6	109	27.0

* Heatwaves are defined as 3 or more consecutive days of mean temperature above the 90th percentile in summer months (May–September).

**Table A3:** Relative Risks (and 95% confidence intervals) of work-related injuries in the agricultural sector for increases in daily mean temperature between 75th to 99th percentile (period 2014–2018), by region.

Regions	RR	95% CI	
Piemonte	1.16	1.13	1.19
Lombardia	1.23	1.21	1.26
Trentino-Alto Adige	1.17	1.12	1.23
Veneto	1.16	1.12	1.20
Friuli-Venezia Giulia	0.90	0.86	0.95
Liguria	1.17	1.11	1.23
Emilia-Romagna	1.22	1.17	1.27
Toscana	1.06	1.00	1.11
Umbria	0.97	0.89	1.07
Marche	1.07	1.01	1.13
Lazio	1.34	1.28	1.40
Abruzzo	1.29	1.23	1.35
Molise	1.15	1.07	1.23
Campania	1.00	0.97	1.04
Puglia	1.14	1.09	1.20
Basilicata	1.15	1.08	1.23
Calabria	0.97	0.93	1.01
Sicilia	1.09	1.05	1.13
Sardegna	1.29	1.24	1.34

**Table A4:** Relative Risks (and 95% confidence intervals) of work-related injuries in the agricultural sector for increases in daily mean temperature between 75th to 99th percentile (period 2014–2018), by working process.

Working Process *	RR	95% CI	
Crop production and harvesting	0.92	0.60	1.41
Plant breeding	0.97	0.67	1.39
Livestock farming and breeding	1.11	0.85	1.47
Land preparation	1.18	1.08	1.30
Auxiliary preparation	1.07	0.77	1.49
Forestry	1.67	0.97	2.86
Other	1.16	1.05	1.27

* Crop production and harvesting: Harvesting, Cutting, Reaping, Threshing; Plant breeding: Seeding, Stratification, Planting; Livestock farming and breeding: Farming, Insemination, Milking, Shearing; Land preparation: Ploughing, Tillage, Drainage, Fertilization; Auxiliary preparation: Mechanical activities, Woodworking, Cleaning, Surveillance Forestry: Cutting down tall trees, Cutting of coppice, Cutting of plants at the height of the stump or collar, First processing of lumber on the spot; *Other*: Other preparations before harvesting, Different activities of reclamation, Special plantations, Further preparations after seeding.
